# The Mechanism by Which Amentoflavone Improves Insulin Resistance in HepG2 Cells

**DOI:** 10.3390/molecules21050624

**Published:** 2016-05-13

**Authors:** Xiaoke Zheng, Yingying Ke, Aozi Feng, Peipei Yuan, Jing Zhou, Yang Yu, Xiaolan Wang, Weisheng Feng

**Affiliations:** 1 School of Pharmacy, Henan University of Traditional Chinese Medicine, Zhengzhou 450046, China; Zhengxk.2006@163.com (X.Z.); keyingying1988@sina.com (Y.K.); 15136153630@163.com (P.Y.); zhoujj1988326@sina.com (J.Z.); ku_rong@126.com (Y.Y.); wxl_325@163.com (X.W.); 2 Center for Artery Atheroscerosis Research, Havana University of Medical Sciences, Havana 999075, Cuba; fengaozi1987@sina.com; 3 Collaborative Innovation Center for Respiratory Disease Diagnosis and Treatment and Chinese Medicine Development of Henan Province, Henan University of Traditional Chinese Medicine, Zhengzhou 450046, China

**Keywords:** AME, insulin resistance, PI3K-Akt, glucose metabolism, inflammatory cytokines

## Abstract

*Background*: The aim of this study was to explore the mechanism by which amentoflavone (AME) improves insulin resistance in a human hepatocellular liver carcinoma cell line (HepG2). *Methods*: A model of insulin resistant cells was established in HepG2 by treatment with high glucose and insulin. The glucose oxidase method was used to detect the glucose consumption in each group. To determine the mechanism by which AME improves insulin resistance in HepG2 cells, enzyme-linked immunosorbent assay (ELISA) and western blotting were used to detect the expression of phosphatidyl inositol 3-kinase (PI3K), Akt, and pAkt; the activity of the enzymes involved in glucose metabolism; and the levels of inflammatory cytokines. *Results*: Insulin resistance was successfully induced in HepG2 cells. After treatment with AME, the glucose consumption increased significantly in HepG2 cells compared with the model group (MG). The expression of PI3K, Akt, and pAkt and the activity of 6-phosphofructokinas (PFK-1), glucokinase (GCK), and pyruvate kinase (PK) increased, while the activity of glycogen synthase kinase-3 (GSK-3), phosphoenolpyruvate carboxylase kinase (PEPCK), and glucose-6-phosphatase (G-6-Pase) as well as the levels of interleukin-6 (IL-6), interleukin-8 (IL-8), tumor necrosis factor-α (TNF-α), and C reactive protein (CRP) decreased. *Conclusions*: The mechanism by which treatment with AME improves insulin resistance in HepG2 cells may involve the PI3K-Akt signaling pathway, the processes of glucose oxygenolysis, glycogen synthesis, gluconeogenesis and inflammatory cytokine expression.

## 1. Introduction

With the rapid development of the social economy, the incidence of metabolic diseases such as obesity, diabetes, and metabolic syndrome has increased. To date, many studies have investigated the pathogenesis of these diseases in which insulin resistance plays an important role [1].

Insulin is the only hypoglycemic hormone in the body. Defects in insulin signal transduction lead to insulin resistance, which is the main cause of metabolic diseases [2,3]. The liver is the main tissue that supplies energy to the body. Insulin resistance in the liver will induce substance and energy dysmetabolism. Glucose metabolism is especially affected. Liver sensitivity to insulin is important for the stabilization of blood sugar levels. Under normal circumstances, the main sources and consumers of blood glucose exist in a dynamic balance to ensure the stability of blood glucose levels. In the event of insulin resistance in the liver, the liver sensitivity to insulin becomes low and blood glucose increases. Under these circumstances, the PI3K-Akt signal transduction pathway inhibits glucose metabolism. Other downstream metabolic and inflammatory pathways are also inhibited [4]. During glucose metabolism, PFK-1, GCK, and PK play vital roles in glucose oxidation. The main function of PFK-1 is the catalytic conversion of 6-phosphoric acid fructose to 1,6-diphosphate fructose; the primary function of GCK is the metabolism of glucose to 6-phosphoric acid and glucose; and the role of PK is to convert phosphoenolpyruvic acid to pyruvic acid and ATP. GSK-3 phosphorylates glycogen synthase to inhibit its activity; PEPCK plays an important role in the catalysis of oxaloacetic acid to phosphoenolpyruvate; and G-6-Pase is the rate-limiting enzyme in the metabolism of glucose 6-phosphate for the production of glucose [5]. If there are dysfunctions in the activities of these glucose metabolic enzymes, the body will fail to regulate increased blood glucose levels [6].

Many studies suggested that insulin resistance is also a process of chronic inflammation and that interactions exist between the inflammation and insulin signaling pathways. Inflammatory cytokines such as IL-6, IL-8, TNF-α, and CRP can impact processes downstream of the insulin signal transduction pathway. For example, when TNF-α interacts with its receptors, a series of processes are activated to interfere with the ability of insulin to interact with the IRS. The PI3K-Akt pathway inhibits the activity of the glucose transporter, and glycogen synthesis decreases. In contrast, once the inflammatory pathways are suppressed, the inhibition of the PI3K-Akt pathway is reversed, thus promoting glucose metabolism. In addition, the inflammatory pathways are activated, and the levels of IL-6, IL-8, TNF-α, and CRP increase significantly [7,8]. As a result, the body secretes more insulin to maintain stable blood glucose levels. This in turn stimulates the liver to increase the body’s insulin resistance and decreases the ability of the body to regulate its blood glucose levels.

*Selaginella Tamariscina (Beauv.) Spring*, which was first recorded in ‘Shen Nong Ben Cao Jing’ (a classical traditional Chinese medicine book) approximately 1700 years ago, is used in folk medicine for the treatment of various diseases including amenorrhoea, dysmenorrhea, and chronic hepatitis. It was found in our previous study that the ethyl alcohol (EtOH) extract from *S. tamariscina* had a hypoglycemic effect. Further analysis revealed that the active compounds of the extract were total flavonoids [9,10,11]. Twelve compounds were isolated from the extract, and 10 of them were identified. AME was found to be the main component of the total flavonoids [12,13]. A review of the literature revealed that AME has PTP1B and alpha-glucosidase inhibitory activity [14]. The identification of the anti-hypoglycemic properties of AME prompted us to evaluate the mechanism by which AME improves insulin resistance in HepG2 cells.

## 2. Results

### 2.1. Subsection

#### 2.1.1. Glucose Consumption in Each Group of Cells

As shown in Table 1, the glucose consumption was significantly decreased in the model group (MG) compared with the normal control group (NC) (*p* < 0.05). The glucose consumption was significantly increased in the metformin group (MF) and in the AMEI, AMEII, and AMEIII groups compared with the MG (*p* < 0.01 or *p* < 0.05). There was no difference between the MF and AME groups (*p* > 0.05).

#### 2.1.2. The Impact of AME on PFK-1, GCK, and PK Activity

As shown in Table 2, the activities of PFK-1, GCK, and PK were significantly decreased in the MG compared with the NC group (*p* < 0.01 or *p* < 0.05). The activities of PFK-1, GCK, and PK were significantly increased in the MF, AMEI, AMEII, and AMEIII groups compared with the MG (*p* < 0.01 or *p* < 0.05). The activity of GCK was significantly increased in the AMEII group compared with the MF group (*p* < 0.05), but there was no significant increase in the AMEI and AMEIII groups compared with the MF group (*p* > 0.05). The activities of PFK-1 and PK were not significantly increased compared with the MF group (*p* > 0.05).

#### 2.1.3. The Impact of AME on the Activity of GSK-3

As shown in Table 3, the activity of GSK-3 was significantly increased in the MG group compared with the NC group (*p* < 0.01). The activity of GSK-3 was significantly reduced in the MF, AMEI, AMEII, and AMEIII groups compared with the MG (*p* < 0.01 or *p* < 0.05). There was no difference between the MF and AME groups (*p* > 0.05).

#### 2.1.4. The Impact of AME on the Activities of PEPCK and G-6-Pase

As shown in Table 4, the activities of PEPCK and G-6-Pase were significantly increased in the MG compared with the NC group (*p* < 0.01 or *p* < 0.05).

The activities of PEPCK and G-6-Pase were significantly reduced in the MF, AMEI, AMEII, and AMEIII groups compared with the MG (*p* < 0.05). There was no difference between the MF and AME groups (*p* > 0.05).

#### 2.1.5. The Impact of AME on the Levels of IL-6, IL-8, TNF-α, and CRP

As shown in Table 5, the levels of IL-6, IL-8, TNF-α, and CRP were significantly elevated in the MG compared with the NC group (*p* < 0.01). The levels of IL-6, IL-8, TNF-α, and CRP were significantly reduced in the MF, AMEI, AMEII, and AMEIII groups compared with the MG (*p* < 0.01 or *p* < 0.05). The levels of IL-6, IL-8, TNF-α, and CRP were significantly increased in the AMEI and AMEIII groups compared with the MF group (*p* < 0.05), but there was no significant increase in the AMEII group compared with the MF group (*p* > 0.05).

#### 2.1.6. The Effect of AME on the PI3K, Akt, and pAkt Proteins

**Figure 1:**
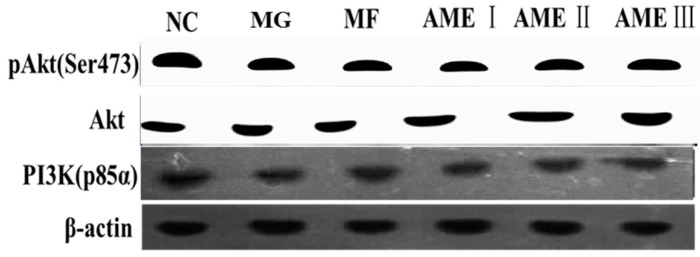
The effect of AME on pAkt, Akt, PI3K proteins (*n* = 3).

**Figure 2:**
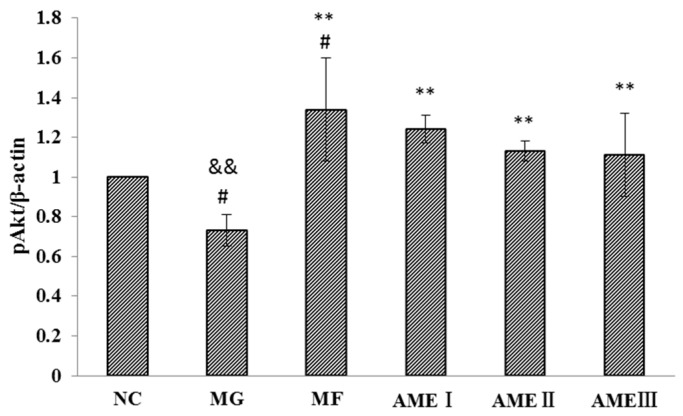
The effect of AME on pAkt (*n* = 3). ^#^: *p* < 0.05 *vs.* the NC; **: *p* < 0.01 *vs.* the MG; ^&&^: *p* < 0.01 *vs.* the MF.

**Figure 3:**
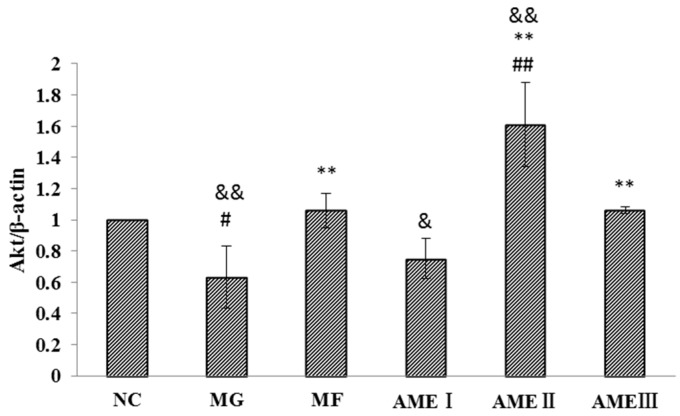
The effect of AME on Akt (*n* = 3). ^#^: *p* < 0.05 *vs.* the NC; ^##^: *p* < 0.01 *vs.* the NC; ^**^: *p* < 0.01 *vs.* the MG; ^&^: *p* < 0.05 *vs*. the MF; ^&&^: *p* < 0.01 *vs*. the MF.

**Figure 4:**
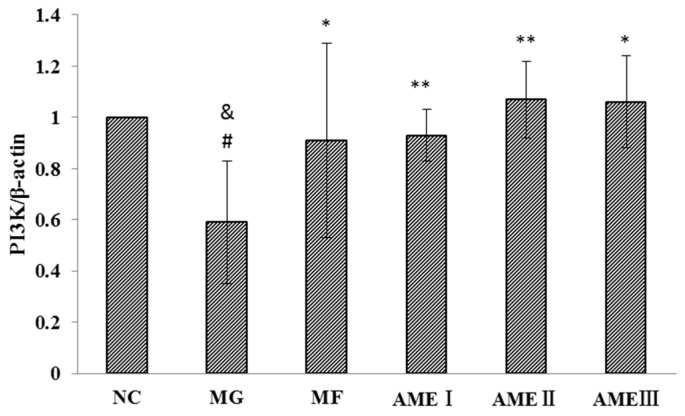
The effect of AME on PI3K (*n* = 3). ^#^: *p* < 0.05 *vs.* the NC; ^*^: *p* < 0.05 *vs.* the MG; ^**^: *p* < 0.01 *vs.* the MG; ^&^: *p* < 0.05 *vs.* the MF.

As shown in Figure 1, Figure 2, Figure 3 and Figure 4, the expression of the PI3K, Akt, and pAkt proteins was significantly decreased in the MG compared with the NC group (*p* < 0.05). The expression of the PI3K, Akt, and pAkt proteins was significantly increased in the MF, AMEI, AMEII, and AMEIII groups compared with the MG (*p* < 0.01 or *p* < 0.05). The expression of PI3K and pAkt was not significantly changed in the AME groups compared with the MF group (*p* > 0.05). The expression of Akt was significantly increased in the AMEII group compared with the MF group (*p* < 0.05).

## 3. Discussion

Insulin is the only hormone that can reduce blood sugar levels in the body. Normally, insulin plays a vital role in lowering blood sugar levels by activating the PI3K-Akt insulin signal transduction pathway [15]. Insulin and insulin receptor form an insulin-insulin receptor complex to activate PI3K. Activated PI3K can catalyze the hydroxylation of phosphatidylinositol-4,5-bisphosphate (PIP2) to form phosphatidylinositol-3,4,5-trisphosphate (PIP3). Upon interacting with the PH domain of Akt, the PIP3-Akt complex localizes to the membrane where cytoplasmic protein kinase B1 (PKB1) interacts with PIP3 through its PH domain. Upon activation of PKB1, Akt is phosphorylated on Thr308. Akt is also phosphorylated on Ser473 by protein kinase B2 (PKB2). Akt becomes activated through these two processes. Activated Akt promotes the translocation of glucose transporter (GLUT) to the cell membrane where it facilitates the entry of extracellular glucose into the cell. At the same time, the activated PI3K-Akt signal transduction pathway activates glucose metabolism and inhibits inflammatory processes [16]. Once insulin resistance begins, the insulin sensitivity of target cells decreases, and the insulin signal transduction pathway becomes disordered [17]. The effect of insulin is reduced, and the regulation of the body’s blood sugar levels is hindered. The body secretes more insulin to regulate blood sugar levels, and excess insulin stimulates the liver, making it less sensitive to insulin. This study aimed to investigate the mechanism by which AME improves insulin resistance in HepG2 cells.

The glucose consumption rate (GC/MTT) indicates the ability of cells to uptake glucose. The glucose oxidase method was used to measure the glucose concentration in each group. The results showed that the GC/MTT ratio decreased in the MG compared with the NC group. After treatment with AME, the GC/MTT ratio was significantly increased in the MF, AMEI, AMEII, and AMEIII groups compared with the MG. There was no difference between the MF and AME groups. Previous studies conducted in our laboratory showed that AME can significantly improve the glucose processing capacity and the insulin resistance of HepG2 cells [18]. The results of our present study are consistent with these earlier observations.

The extract and monomeric compounds of *Selaginella Tamariscina (Beauv.) Spring* have been studied in our laboratory. Investigating the impact of AME on insulin resistance in HepG2 cells is the current focus of our lab. Liu found that amentoflavone protects vascular endothelial cells and down-regulates downstream inflammation and oxidative damage factors through the NF-κB signaling pathway [19]. The doses of AME tested were 2, 4, 6, and 8 μg/mL, and the optimal dose of AME was 6 μg/mL. We took this information into account before we started our research. Because of the differences between our study and that of Liu, we also tested the effect of AME on insulin resistance. The results showed that 13.94 × 10^−3^ mmol/L was the best dose. The doses of AME and MF were selected because they improved insulin resistance in HepG2 cells to the greatest extent. We compared the results generated by the optimum doses of AME and MF. We found differences between AME and commonly used clinical drugs. This provided the basis for further research.

As mentioned previously, the PI3K-Akt signaling pathway plays an important role in improving insulin resistance [20,21]. The impairment of PI3K-Akt-mediated insulin signaling induces insulin resistance [22]. Kang found that the expression of PI3K was decreased in a diabetes model. A gold capsule could improve the expression of PI-3K and GLUT4 [23]. Dai found that the expression of Akt mRNA was significantly decreased in the adipose tissue of diabetic rats. Liuweidihuang tang and alcohol extract could improve the PI3K/Akt signaling pathway-mediated regulation of blood glucose in type 2 diabetic rats [24]. Liu found that the expression of p-Akt was decreased in a model of insulin resistance. Jinlida could effectively reduce insulin resistance and improve glucose and lipid metabolic disorders in rats fed high-fat diets. The possible molecular mechanism might involve the regulation of the PI3K/AKT signaling pathway [25]. In our research, the expression of PI3K, Akt, and pAkt was decreased in the MG. After treatment with MF and AME, the expression of PI3K, Akt, and pAkt was significantly reduced. The experimental results showed that the expression of PI3K, Akt, and pAkt proteins in the MG was significantly decreased compared with the NC group, and the expression of the PI3K, Akt, and pAkt proteins was significantly increased in the MF, AMEI, AMEII, and AMEIII groups compared with the MG after treatment with AME. The results conclusively show that that AME can improve insulin resistance in HepG2 cells and that its mechanism may be related to the regulation of the PI3K-Akt signal transduction pathway. In the analysis of the experimental data, the first thing we considered was the difference between the normal group and the model group. We found that the expression of PI3K and Akt was decreased in the model group. We then focused on the effects of AME and found that the expression of PI3K and Akt increased. The results also showed that the total levels of Akt in the AMEII-treated cells were almost double in comparison to the control cells, whereas the PI3K values in the AMEII-exposed cells were similar to those in untreated cells. We hypothesized that the PI3K/Akt signaling pathway is the target of AME, but that AME is more inclined to promote Akt protein expression. Variations in total AKT values were higher than the variations in pAKT levels. This may be because AME can not only improve the expression of Akt, but can also promote the phosphorylation of Akt. In agreement with this, we only detected Akt1; the other subtypes were not detected. Much anticipated future studies will measure the level of Akt mRNA.

The stability of the body’s blood glucose levels is closely related to the insulin signal transduction pathway [26]. Glucose metabolism is a large and complex system. PFK-1, GCK, PK, GSK-3, PEPCK, and G-6-Pase play vital roles in glucose metabolism. Our previous research did not investigate the enzymes involved in the process of glucose oxidation decomposition. We searched the relevant literature and found that there is no system for detecting these enzymes. During glucose oxidation [27], there are a few rate-limiting, irreversible reactions in which catalytic enzymes such as PFK-1, GCK, and PK play vital roles. The experimental results showed that the activities of PFK-1, GCK, and PK were significantly decreased in the MG compared to the NC group. After treatment with AME, the activities of PFK-1, GCK, and PK were significantly increased in the MF, AMEI, AMEII, and AMEIII groups compared with the MG.

Glycogen synthesis plays a key role in reducing postprandial hyperglycemia. Glycogen synthase is the rate-limiting enzyme in this process. The main role of GSK-3 is to phosphorylate glycogen synthase to inhibit its ability to synthesize glycogen [28]. Gong found that low-power laser irradiation (LPLI) could promote glucose uptake and glycogen synthesis and improve the insulin resistance of skeletal muscle by activating the PI3-K/Akt2/GLUT4 signaling pathway [29]. The experimental results showed that the expression of GSK-3 protein in the MG was significantly increased compared with the NC group and that after treatment with AME, GSK-3 protein was significantly increased in the MF, AMEI, AMEII, and AMEIII groups compared with the MG.

Gluconeogenesis is the process by which non-sugar substances are transformed into glucose. Gluconeogenesis provides supplemental glucose under starvation conditions. PEPCK plays an important role in the catalysis of oxaloacetic acid to phosphoenolpyruvate [30]. With regard to gluconeogenesis, Zhang found that the activity of PEPCK was significantly increased in the HepG2 model of insulin resistance. Gegen Qinlian Tang-containing serum reduced the activity of PEPCK and improved gluconeogenesis [31]. G-6-Pase is the rate-limiting enzyme in the metabolism of glucose 6-phosphate to glucose [32]. The experimental results showed that the expression of PEPCK and G-6-Pase was significantly increased in the MG and that they were significantly reduced after treatment with AME.

Many studies suggested that insulin resistance is a process of chronic inflammation and that the inflammatory and PI3K-Akt signaling pathways interact to some degree [33]. *In vivo*, the presence of normal amounts of inflammatory cytokines does not cause damage. However, once insulin resistance arises, the inflammatory pathways are activated, and the levels of IL-6, IL-8, TNF-α, and CRP increase significantly and produce different degrees of injury in the body [34]. Inflammatory cytokines can impact the processes of the insulin signal transduction pathway. With the induction of inflammatory cytokines, insulin resistance is induced and aggravated [35]. Inflammatory cytokines such as IL-6, IL-8, TNF-α, and CRP can impact the processes of the insulin signal transduction pathway. After TNF-α interacts with its receptors, a series of processes are activated that interfere with the ability of insulin to interact with the IRS. The PI3K-Akt pathway is inhibited along with the activity of the glucose transporter and glycogen synthesis. However, once the inflammatory pathways are suppressed, the inhibition of the PI3K-Akt pathway is reversed, and the related processes of glucose metabolism are promoted. Lin found that type 2 diabetes mellitus is a chronic low-grade inflammatory disease. It is often accompanied by higher levels of inflammatory markers that are important for the regulation of the inflammatory process [36]. From the experimental results, we found that the levels of IL-6, IL-8, TNF-α, and CRP were significantly higher in the MG compared with the NC group and that the levels of IL-6, IL-8, TNF-α, and CRP were significantly reduced in the MF, AMEI, AMEII, and AMEIII groups compared with the MG. The effects of AMEI, AMEII, and AMEIII were not dose-dependent. Overall, the effects were the greatest in the AMEII group. Why was the AMEII dose more effective than the AMEI and AMEIII doses in the reduction of pro-inflammatory protein levels? The studies regarding the PI3K-Akt signaling pathway revealed that the expression of PI3K and Akt in the AMEII group was higher than in the AMEI and AMEIII groups. We can surmise that AMEII has a more pronounced effect on the PI3K-Akt signaling pathway than AMEI and AMEIII and that this improves the effect on the inflammatory pathways. Thus, the AMEII dose was more effective than AMEI and AMEIII in reducing pro-inflammatory protein levels.

In a preliminary study conducted in our laboratory, Li found that the total flavonoids of *Selaginella tamariscina* (Beauv.) Spring (TFST) have potent anti-hypoglycemic effects [11]. Zhang proved that TFST promotes the long-term stabilization of blood glucose levels and controls lipids and lipoproteins by regulating the secretion of insulin and glucagon in the pancreas. The mechanism might involve its ability to improve liver structure and increase the protein expression of PPAR-γ and IRS-1 [12]. Wang found that TFST reduces high blood glucose levels but has no effect on normal blood glucose levels. Lipidosis and hepatic steatosis were ameliorated by TFST via a mechanism involving the expression of visfatin in adipose tissue [13]. Further studies showed that the main component of TFST is AME. Su demonstrated that AME can ameliorate glucose and lipid metabolism disorders without causing significant injury to the liver and kidney. AME can regulate the activities of the enzymes involved in glucose metabolism. AME can activate the PI3K-Akt pathway; this is probably one of the underlying mechanisms responsible for its antidiabetic effect [14]. Combined with the previous results, our research put the focus on the PI3K-Akt pathway. The results showed that the mechanism by which AME improves insulin resistance in HepG2 cells may be related to the PI3K-Akt signaling pathway, which promotes glucose oxygenolysis and glycogen synthesis and inhibits gluconeogenesis and the level of inflammatory cytokines.

## 4. Materials and Methods

### 4.1. Preparation of AME

Dried *Selaginella* was purchased from the Ben Cao Guo Yao Tang LLC (Zhengzhou, China) and identified by Chen Sui-qing of the Henan University of Traditional Chinese Medicine. The purity of the AME prepared in our laboratory is more than 98% as determined by UV spectrophotometry. AME (1.00 mg) was dissolved in 10 μL DMSO and added to 10 mL DMEM containing 0.2% BSA. The solution was stored in the 4 °C refrigerator.

### 4.2. Cell Culture

HepG2 (human hepatocellular liver carcinoma) cells were a gift from Qiujun Lv of the Radiation Academy of Military Medical Sciences (Beijing, China). The cells were cultured in Dulbecco’s modified Eagle medium (DMEM, Gibco, Pittsburgh, PA, USA, 4.5 g/L) supplemented with 10% fetal bovine serum, 100 kU/L penicillin, and 100 kU/L streptomycin at 37 °C in an atmosphere of 5% CO_2_.

### 4.3. Reagents and Instruments

DMEM (Gibco); bovine serum albumin (BSA) (Gibco); trypsin (Gibco); fetal bovine serum (Gibco); insulin (American Sigma Company, Santa Clara, CA, USA); glucose detection reagent box (Biosino Biotechnology Co., Ltd., Beijing, China); glucokinase (GCK), 6-phosphofructokinase (PFK-1), pyruvate kinase (PK), glycogen synthase kinase-3 (GSK-3), phosphoenolpyruvate carboxylase kinase (PEPCK), glucose-6-phosphatase (G-6-Pase), IL-6, IL-8, TNF-α, and CRPELISA kits (all purchased from R & D Systems, Minneapolis, MN, USA); total protein extraction and BCA protein assay kits (Applygen Technologies, Inc., Beijing, China); PI3K, Akt, and pAkt polyclonal antibodies (Abcam Inc., Cambridge, UK); rabbit anti-mouse β-actin (ab75186, Abcam Inc.); pre-stained marker (J114, H30315, FERMENTAS Inc., Burlington, MA, USA); enhanced chemiluminescence (ECL) kit (Beijing Kang Century Biotech Co., Ltd., Beijing, China); methanol (Tianjin four chemical reagent company, Tianjin, China); ultra-pure water (611VF Sartorius, Gottingen, Germany); PBS buffer; other commercially available chemical reagents.

Carbon dioxide incubator (Shanghai STIK, Shanghai, China); clean bench (Jiangsu Sujing Group Co., Ltd., Suzhou, China); iMARK TM microplate reader (Bio-Rad, Hercules, CA, USA); Arium VF 611 super combination ultrapure water (Sartorius AG, Germany); 5804R small, high-speed, low-temperature refrigerated centrifuge (Eppendorf AG, Hamburg, Germany); AB204-N millionth precision analytical balance (Mettler Toledo International Co., Ltd., Zurich, Switzerland); DYY-24DN electrophoresis apparatus (Beijing, China); DYCZ-24DN-type vertical slab electrophoresis apparatus; semi transmembrane instrument (GE); G:BOX gel imager (Syngene, Cambridge, MA, USA); inverted microscope (Nikon, Tokyo, Japan); PB-10 pH meter (Sartorius AG, Germany); dishes, 96-well culture plates, and cryopreservation tubes (Corning, Inc., New York, NY, USA).

### 4.4. Methods

#### 4.4.1. Generation of an Insulin Resistance Model

HepG2 cells were seeded in 96-well plates. The density of the cells in each plate was 2 × 10^4^ cells/mL. The cells were cultured at 37 °C in an atmosphere of 5% CO_2_. After the cells became adherent, we removed the original medium and washed the cells twice with DMEM. The medium of the normal control group was replaced with DMEM containing 10% FBS. The medium of the model group was replaced with DMEM containing 10% FBS and 10^−7^ mol/L insulin. After the cells were cultured for 48 h, we detected the glucose consumption rate in each group to validate the model [37].

#### 4.4.2. The Experimental Groups

There were six groups in our study: the normal control group (NC), the model group (MG) (treated with 10^−7^ mol/L insulin and cultivated for 48 h), the metformin group (MF) (treated with 0.1 mmol/L metformin), and three amentoflavone groups (AMEI, AMEII, and AMEIII, which were treated with 9.30 × 10^−3^ mmol/L, 13.94 × 10^−3^ mmol/L, and 18.59 × 10^−3^ mmol/L AME, respectively). The AME groups were treated with AME for 36 h following culture for 48 h in DMEM containing a high dose of insulin.

#### 4.4.3. Measurement of the Glucose Consumption Rate

HepG2 cells were seeded in 96-well plates in DMEM supplemented with 10% (V/V) FBS. The density of the cells in each plate was 2 × 10^4^ cells/mL. We removed the original medium when the cells became adherent and replaced it with DMEM containing 10^−7^ mol/L insulin. The model of insulin resistance was established after 48 h [37]. We added drug-free or drug-containing DMEM containing 0.2% BSA and then divided the cells into the normal group, the model group, and the different AME-administered groups. We then used the glucose oxidase method to detect glucose consumption (GC) levels after 36 h. The glucose oxidase method was used to detect the glucose concentration in each group. Glucose oxidase catalyzes the breakdown of glucose to glucose acid and hydrogen peroxide. Peroxidase then catalyzes the formation of red quinone imide in a reaction involving hydrogen peroxide, 4-aminoantipyrine, and phenol. The absorbance of red quinone imide at a wavelength of 490 nm is proportional to the concentration of glucose [37]. Finally, we added 20 μL MTT solution (12.08 × 10^−3^ mmol/L) to each well and cultured the cells for 4 h in the incubator. After removing the medium from each well, we added 150 μL DMSO and shocked the cells for 10 min to dissolve the crystals. The absorbance (A) was measured at a wavelength of 490 nm using a microplate reader. The glucose consumption rate was calculated using the equation GC/MTT.

#### 4.4.4. ELISA

For the ELISA assay, a 100 mm × 20 mm Petri dish was used. The density of cells in each dish was 2 × 10^5^ cells/mL. After treatment with AME for 36 h, we collected the culture medium from each petri dish and centrifuged it. The supernatants were collected and used to detect the levels of IL-6, IL-8, TNF-α, and CRP according to the respective manufacturer’s instructions. The cells in each group were also collected to extract total protein according to the manufacturer’s instructions. The cells were collected in 2 mL centrifuge tubes, and 10 mL protease inhibitor and 10 mL protein phosphatase inhibitor were added. Then, 0.5 mL lysis buffer was added to the centrifuge tube. The cells were resuspended and placed at 4 °C for 2 min. Then, 1 mL extraction reagent was added. The mixture was blended and placed at 4 °C for 10 min. The solution was divided into two phases after centrifuging at 10,000× *g* for 10 min. The solid between the two phases was total protein. One milliliter ethanol was added to the centrifuge tube. Total protein was purified after centrifuging at 10,000× *g* for 3 min. The solid precipitate was the total protein. The solid protein was dissolved in a 2% SDS buffer solution and boiled at 95 °C for 10 min. The precipitate was dissolved at room temperature for 20–60 min. The supernatant, which was the protein solution, was collected after centrifugation. The protein solution was used in western blotting experiments after its concentration was determined using the BCA protein assay kit. After dissolving the protein and measuring its concentration, we used the extract to determine the activities of PFK-1, GCK, PK, GSK-3, PEPCK, and G-6-Pase.

#### 4.4.5. Western Blot Analysis

In the western blotting experiments, total protein was extracted according to the manufacturer’s instructions. The extraction method for total protein was same as that described above for the ELISA. The protein samples were separated by SDS-PAGE. Forty micrograms of each protein sample, an amount that was chosen in a preliminary experiment, was loaded into the gel and transferred to a PVDF membrane. Then, we used non-fat milk to block the membrane for 2 h at room temperature and to blot with a primary antibody overnight at 4 °C. After being washed in TBST buffer, the bound proteins were detected with a secondary antibody for 1 h at room temperature. The intensity of the target proteins and the reference protein was quantified using Gene Tools.

### 4.5. Statistical Analyses

All of the data are expressed as the mean ± standard deviation. Statistical significance was assessed in comparison with the respective control for each experiment using one-way ANOVA. *p* values < 0.05 were regarded as statistically significant.

## 5. Conclusions

The mechanism by which treatment with AME improves insulin resistance in HepG2 cells may involve the PI3K-Akt signaling pathway, the processes of glucose oxygenolysis, glycogen synthesis, gluconeogenesis and inflammatory cytokine expression.

## Figures and Tables

**Table 1:** Glucose consumption ($\bar{x}$ ± s, *n* = 3).

Group	Glucose Consumption (GC/MTT)
NC	3.85 ± 0.75
MG	2.67 ± 0.94 ^#,&&^
MF	3.96 ± 0.53 **
AMEI	3.70 ± 0.34 *
AMEII	3.81 ± 0.07 *
AMEIII	3.66 ± 0.41 *

^#^: *p* < 0.05 *vs.* the NC; *: *p* < 0.05 *vs.* the MG; **: *p* < 0.01 *vs.* the MG; ^&&^: *p* < 0.01 *vs.* the MF.

**Table 2:** The impact of AME on PFK-1, GCK, and PK activity o ($\bar{x}$ ± s, *n* = 3).

Group	PFK-1 (U·L^−1^)	GCK (U·L^−1^)	PK (mU·L^−1^)
NC	1485.91 ± 97.55	230.9 ± 5.69	221.94 ± 8.36
MG	1111.60 ± 122.67 ^##,&&^	154.48 ± 29.00 ^##,&&^	189.10 ± 5.50 ^#^
MF	1464.34 ± 134.67 **	221.52 ± 16.74 **	216.53 ± 18.26 *
AMEI	1458.57 ± 171.74 *	259.05 ± 22.75 **	218.09 ± 19.26 *
AMEII	1562.65 ± 171.74 **	267.10 ± 11.38 **^,&^	218.05 ± 23.86 *
AMEIII	1441.22 ± 196.27 *	238.94 ± 5.69 **	217.58 ± 18.49 *

^#^: *p* < 0.05 *vs.* the NC; ^##^: *p* < 0.01 *vs.* the NC; *: *p* < 0.05 *vs.* the MG; **: *p* < 0.01 *vs.* the MG; ^&^: *p* < 0.05 *vs.* the MF; ^&&^: *p* < 0.01 *vs.* the MF.

**Table 3:** The impact of AME on the activity of GSK-3 ($\bar{x}$ ± s, *n* = 3).

Group	GSK-3 (U·L^−1^)
NC	131.69 ± 1.24
MG	142.43 ± 6.20 ^##^
MF	134.55 ± 3.72 *
AMEI	131.09 ± 3.14 **
AMEII	131.80 ± 6.11 **
AMEIII	134.66 ± 3.63 *

^##^: *p* < 0.01 *vs.* the NC; *: *p* < 0.05 *vs.* the MG; **: *p* < 0.01 *vs.* the MG.

**Table 4:** The impact of AME on the activities of PEPCK and G-6-Pase ($\bar{x}$ ± s, *n* = 3).

Group	PEPCK (IU·L^−1^)	G-6-Pase (mIU·mL^−1^)
NC	38.50 ± 2.40	994.99 ± 62.15
MG	49.22 ± 3.34 ^##,&^	1412.45 ± 93.22 ^#^
MF	41.27 ± 2.75 *	1082.87 ± 62.15
AMEI	40.92 ± 6.25 *	1024.28 ± 207.67 *
AMEII	39.19 ± 4.52 *	1060.90 ± 93.22 *
AMEIII	40.57 ± 4.19 *	1068.23 ± 166.37 *

^#^: *p* < 0.05 *vs.* the NC; ^##^: *p* < 0.01 *vs.* the NC; *: *p* < 0.05 *vs.* the MG; ^&^: *p* < 0.05 *vs.* the MF.

**Table 5:** The impact of AME on the levels of IL-6, IL-8 and TNF-α, CRP ($\bar{x}$ ± s, *n* = 3).

Group	IL-6(ng·L^−1^)	IL-8(ng·L^−1^)	TNF-α (pg·mL^−1^)	CRP (μg·L^−1^)
NC	6.24 ± 4.05	247.06 ± 53.08	331518.25 ± 24999.24	494.72 ± 91.22
MG	36.48 ± 8.01 ^##,&&^	707.00 ± 45.07 ^##,&&^	492280.22 ± 68203.98 ^##,&&^	1461.54 ± 249.72 ^##,&&^
MF	11.03 ± 2.54 **	247.06 ± 45.07 **	333865.14 ± 19046.96 **	540.45 ± 203.65 **
AMEI	34.83 ± 5.21 ^##,&&^	694.31 ± 125.63 ^##,&&^	433216.82 ± 28692.27 ^##,^*^,&&^	1161.04 ± 133.35 ^##,^*^,&&^
AMEII	12.91 ± 9.08 **	283.27 ± 99.23 **	371806.53 ± 26416.85 **	622.10 ± 129.63 **
AMEIII	28.66 ± 2.44 ^##^*^&&^	524.61 ± 82.33 ^##,^**^,&&^	436815.38 ± 37085.32 ^##,^*^,&&^	1185.54 ± 161.40 ^##,^*^,&&^

^##^: *p* < 0.01 *vs.* the NC; *: *p* < 0.05 *vs.* the MG; **: *p* < 0.01 *vs.* the MG; ^&&^: *p* < 0.01 *vs.* the MF.

## Abbreviations

The following abbreviations are used in this manuscript:

AME: Amentoflavone
ELISA: Enzyme-linked immunosorbent assay
PI3K: Phosphatidyl inositol 3-kinase
PFK-1: 6-Phosphofructokinase
GCK: Glucokinase
PK: Pyruvate kinase
GSK-3: Glycogen synthase kinase-3
PEPCK: Phosphoenolpyruvate carboxylase kinase
G-6-Pase: Glucose-6-phosphatase
IL-6: Interleukin-6
IL-8: Interleukin-8
TNF-α: Tumor necrosis factor-α
CRP: C reactive protein
NC group: Normal control group
MG: Model group
MF: Metformin group
GC: Glucose consumption
MTT: 3-(4, 5-Dimethylthiazol-2-yl)-2,5-diphenyltetrazolium bromide
GLUT: Glucose transporter
DMSO: Dimethyl sulfoxide
BSA: Bovine serum albumin
PIP2: Phosphatidylinositol-4,5-bisphosphate
PIP3: Hydroxyl to form phosphatidylinositol-3,4,5-trisphosphate
PKB1: Protein kinase B1
PKB2: Protein kinase B2
TFST: Total Flavonoids of *Selaginella tamariscina (Beauv.)* Spring
